# Do hypoxia and L-mimosine modulate sclerostin and dickkopf-1 production in human dental pulp-derived cells? Insights from monolayer, spheroid and tooth slice cultures

**DOI:** 10.1186/s12903-018-0492-8

**Published:** 2018-03-09

**Authors:** Klara Janjić, Barbara Cvikl, Christoph Kurzmann, Andreas Moritz, Hermann Agis

**Affiliations:** 1 Department of Conservative Dentistry and Periodontology, School of Dentistry, Medical University of Vienna, Sensengasse 2a, 1090 Vienna, Austria; 2 Austrian Cluster for Tissue Regeneration, Donaueschingenstr. 13, Vienna, 1200 Austria; 3 Department of Preventive, Restorative and Pediatric Dentistry, University of Bern, Freiburgstrasse 7, Bern, 3010 Switzerland

**Keywords:** Dental pulp, Dickkopf-1, Hypoxia, Hypoxia mimetic agents, Sclerostin, Spheroids

## Abstract

**Background:**

To understand the responses of the dental pulp to hypoxia is of high relevance for regenerative endodontics and dental traumatology. Here, we aimed to reveal the effects of hypoxia and the hypoxia mimetic agent L-mimosine (L-MIM) on the production of sclerostin (SOST) and dickkopf-1 (DKK-1) in human dental pulp-derived cells (DPC).

**Methods:**

DPC in monolayer, spheroid and tooth slice cultures were treated with L-MIM or hypoxia. Resazurin-based toxicity and MTT assays were performed to determine cell viability. mRNA and protein levels of SOST and DKK-1 were measured with quantitative reverse transcription PCR and ELISA, respectively. To validate the hypoxia-like response, SDF-1, VEGF and IL-8 were assessed. In addition Western blots for HIF-1α, HIF-2α and HIF-3α were done.

**Results:**

Cells were vital upon treatment procedures and showed increased levels of HIF-1α, and HIF-2α. In monolayer cultures, mRNA levels of SOST and DKK-1 were downregulated by L-MIM and hypoxia, respectively. A significant downregulation of SOST by hypoxia was found at the protein level compared to untreated cells while the effect on DKK-1 and the impact of L-MIM on SOST and DKK-1 did not reach the level of significance at the protein level. In spheroid cultures, mRNA levels of SOST and DKK-1 were downregulated by L-MIM. A significant downregulation of DKK-1 upon hypoxia treatment was found at the protein level while the impact of hypoxia on SOST and the effect of L-MIM on SOST and DKK-1 did not reach the level of significance. SOST and DKK-1 were also produced in tooth slices, but no pronounced modulation by L-MIM or hypoxia was found. Evaluation of SDF-1, VEGF and IL-8 showed a hypoxia-like response in the culture models.

**Conclusions:**

There is no pronounced influence of hypoxia and L-MIM on DPC viability, SOST and DKK-1 protein production. However, the specific response depends on the culture model and the level of evaluation (mRNA or protein). These results deepen our understanding about the role of hypoxia and the potential impacts of hypoxia-based strategies on dental pulp.

## Background

It is of high relevance for regenerative endodontics and dental traumatology to understand the response of the dental pulp to hypoxia. During tissue regeneration, angiogenesis is an important process in the response of the dental pulp to hypoxia. Clinically applied pulp re-vascularisation treatment requires functional angiogenesis to facilitate the repair of dental pulp [1]. Experimental tissue engineering strategies which rely on cell transplantation also require formation of highly vascularised tissue [2]. Consequently, strategies have been developed to stimulate angiogenesis. Hypoxia-based strategies such as hypoxia conditioning or the application of hypoxia mimetic agents have been developed as new approaches to stimulate regeneration by inducing hypoxia-responsive factors that support angiogenesis and improve cell survival [3]. Hypoxia mimetic agents such as prolyl hydroxylase inhibitors, for example L-mimosine (L-MIM) and deferoxamine, have shown to induce a similar pro-angiogenic response as hypoxia conditioning in dental pulp cells and can induce reparative processes [4–8]. This led researchers to analyse the effects of hypoxia on the dental pulp tissue and its role in regeneration.

Formation of tertiary dentine in hard tissue defects is essential for the success of pulp regeneration strategies. Here, the differentiation of progenitor cells into active dentine-forming odontoblasts is required. This process is regulated by WNT signalling [9, 10] which plays a role in the development [11], homeostasis [12] and regeneration [13] of dental tissues. The application of recombinant WNTs stimulates proliferation and modulates differentiation of odontoblasts and dentine-like tissue formation in vitro and in vivo [9, 11, 14, 15]. The activity of WNTs is tightly controlled by the WNT signalling inhibitors sclerostin (SOST) and dickkopf (DKK)-1. Both inhibitors are considered to be potential targets to treat diseases. Monoclonal antibodies against SOST were evaluated in pre-clinical models for bone regeneration in periodontitis [16] and tested in clinical studies for application in osteoporosis patients [17]. The role of SOST and DKK-1 in the dental pulp and its relevance in pulp healing is unclear. Given, the importance of SOST and DKK-1 in hard tissue formation and associated diseases, and their relevance in regenerative approaches, it is highly pertinent to understand the impact of therapeutic approaches involving application of hypoxia mimetic agents and hypoxia pre-conditioning on the WNT inhibitors.

The response of SOST to hypoxia is diversely discussed. Hypoxia decreases SOST levels in osteoblasts and osteocytes [18]. However, also an increase of SOST upon hypoxia [19] and an increase of DKK-1 in bone marrow-derived mesenchymal stem cells [20] was observed. This modulation of WNT inhibitors, paralleled by a reduction of the osteoblastic differentiation and responsiveness to differentiation factors upon hypoxia was reported [21, 22]. Similarly, low oxygen levels inhibit matrix mineralisation by dental pulp progenitor cells [7, 23]. Also, inhibitory effects on matrix mineralisation were described for the hypoxia mimetic agent L-MIM [7, 23]. Together these data suggest the possibility that hypoxia and hypoxia mimetic agents modulate WNT pathway inhibitors in dental pulp cells. It is however unknown if WNT inhibitors such as SOST and DKK-1 are also modulated in dental pulp cells.

In this study, we hypothesised that hypoxic conditions modulate the expression of the WNT signalling inhibitors SOST and DKK-1. We aimed to reveal the effects of hypoxia and L-MIM on the production of SOST and DKK-1 in human dental pulp-derived cells (DPC). We used 2D monolayer cultures and 3D spheroid cultures of DPC as well as tooth slice cultures to follow our hypothesis. The response to hypoxic conditions were validated by evaluation of hypoxia-inducible factor (HIF)-1α, HIF-2α, and HIF-3α, stromal cell-derived factor (SDF)-1, vascular endothelial growth factor (VEGF) and interleukin (IL)-8.

## Methods

### Human dental pulp-derived cell isolation and culture

DPC were isolated from donor teeth of adult patients with their informed consent at the School of Dentistry, Medical University of Vienna, Vienna, Austria. The protocol was approved by the Ethics Committee of the Medical University of Vienna (#631/2007). Cells that grew out from extracted dental pulp tissue in cell culture medium (αMEM, Gibco, Invitrogen Corporation, Carlsbad, CA, USA) with 10% foetal bovine serum (FBS; LifeTech, Vienna, Austria), 100 U/mL penicillin G, 100 μg/mL streptomycin and 2.5 μg/mL amphotericin B (all Gibco, Invitrogen Corporation, Carlsbad, CA, USA) were further cultured as DPC. DPC consist of a mixed population. As fibroblasts have a higher proliferation rate within the pulp it can be assumed that the DPC population predominantly consists of fibroblasts. A detailed description of the cell isolation procedure has already been published [7]. DPC were incubated in cell culture medium at 37 °C, 5% CO_2_ and 95% atmospheric moisture. All cell culture methods were conducted under sterile conditions. DPC of passages 3–7 were used for the experiments.

### 2D monolayer cell culture

DPC were cultured as a monolayer at a density of 50,000 cells/cm^2^. On the next day cells were treated with L-MIM (1 mM) or hypoxia for 24 h. The concentration choice of L-MIM is based on previous findings in DPC [7, 24]. Hypoxic conditions were induced by culturing cells in a BD GasPak EZ Pouch system (Becton, Dickinson and Company, Franklin Lakes, NJ, USA) as previously described [6, 8, 25]. This treatment procedur mimics severe hypoxia < 1 % O_2_, as also suggested by Agata et al. [26], being biologically relevant [26, 27]. Untreated cells under normoxic conditions are presented as control. Directly after the 24 h treatment period, supernatants were collected for enzyme-linked immunosorbent assays (ELISA) and RNA was isolated from cells. Also viability assays were performed 1 day after treatment.

### 3D spheroid cell culture

3D spheroid cultures of DPC were performed in two different ways. For cell viability experiments, 96 well plates were coated with 1.5% agarose. 30,000 cells/cm^2^ were added to coated plates and left for incubation as described above for 1 day to form DPC spheroids. Cell density was based on previous publications reporting DPC spheroid culturing [28].

For collection of supernatant and RNA isolation, 3D spheroid cultures were created using 3D Petri dishes® (Microtissues, Inc., Providence, RI, USA). Petri dishes® served as a pattern to produce moulds out of agarose with 35 cavities for spheroids. The agarose moulds were conditioned with cell culture medium and placed into 24 well plates where they were filled with cell suspensions of 547,500 cells in drops of 75 μL. Then, wells were filled with 1 mL of cell culture medium and plates were incubated for 24 h as described above.

In both 3D spheroid culture variants, spheroids formed within 1 day. After this, they were treated with L-MIM (1 mM) or hypoxia as described in the 2D model for 24 h. Here, spheroids without treatment and under normoxic conditions served as controls. Directly after the 24 h treatment spheroids were proceeded to RNA isolation, collection of supernatant and viability assays.

### Tooth slice culture

The tooth slice culture was performed as described previously [7, 8, 25, 29]. Dentists at the School of Dentistry in Vienna extracted donor teeth from adult patients with their informed consent as approved by the Ethics Committee of the Medical University of Vienna. Teeth were transferred to cell culture medium without FBS directly after extraction. Attached tissue such as gingiva, was removed and teeth were processed immediately. To produce tooth slices, teeth were cut in transverse plane into 600 μm thick slices containing vital pulp tissue, using a diamond saw (IsoMet® Low Speed Saw, Buehler, Lake Bluff, IL, USA). All these procedures were performed at room temperature. After cutting, gained tooth slices containing the vital pulp tissue were transferred to 24 well plates, covered with culture medium and incubated at 37 °C, 5% CO_2_ and 95% atmospheric moisture. After 2 days, tooth slices were treated with L-MIM (1 mM) or hypoxia for 48 h. Untreated tooth slices under normoxia served as control. Directly after the 48 h treatment period, supernatants were collected and tooth slices were subjected to viability assays or RNA isolation. The absolute protein concentration in the normoxic control group and the treatment groups was normalized to the respective MTT results to correct the volume of metabolic active tissue and is presented relative to the corresponding control as described previously [7, 8, 25, 29].

### Resazurin-based toxicity assay

The resazurin-based toxicity assay (Sigma-Aldrich, Vienna, Austria) was used to determine cell viability in 2D monolayer and 3D spheroid cultures of DPC. In the cultures for this assay, a negative control containing only cell culture medium was added to the experiments. Resazurin solution was added directly after cell treatment. After incubation for 24 h, fluorescence was measured in cell culture supernatants at 590 nm, with an excitation wavelength of 560 nm. Results were normalised to the negative control. These calculated data were normalised to the normoxic control.

### MTT assay

The MTT assay (Sigma-Aldrich) was used to measure cell viability in tooth slices. Two days after treatment, MTT solution (Sigma-Aldrich) was added to tooth slices. After 2 h of incubation, the reaction was stopped with DMSO and optical density of supernatants was measured at 550 nm. Results were normalised to the normoxic control.

### RNA isolation, reverse transcription and quantitative polymerase chain reaction

Total RNA was isolated using an RNeasy Plus Mini Kit (Qiagen, Hilden, NW, Germany), following the manufacturer’s instructions. Additionally, tooth slice culture samples had to be sonicated to separate pulp tissue from dentine and to disrupt the tissue. After RNA isolation the 260 nm / 280 nm ratio as well as the RNA concentration was measured in a photometer. Next, 1 μg RNA in 2D samples and 0.15 μg RNA in 3D samples was used to generate cDNA with the High-Capacity cDNA Reverse Transcription Kit (Thermo Fisher Scientific, Waltham, MA, USA). SOST, DKK-1, SDF-1, VEGF and IL-8 expression levels were measured relative to the reference gene Glycerinaldehyde-3-phosphate dehydrogenase (GAPDH*)* by qPCR. For this, TaqMan© Real-Time qPCR Master Mix (Applied Biosystems, Carlsbad, CA) and TaqMan© assays (Table 1, Thermo Fisher Scientific) for SOST, DKK-1, SDF-1, VEGF and IL-8 were used to amplify cDNA samples.

**Table 1:** TaqMan® Assays (Thermo Fisher Scientific) that were used in qPCR analysis

	Gene name	TaqMan® assay ID
SOST	sclerostin	Hs00228830_m1
DKK-1	dickkopf WNT signaling pathway inhibitor 1	Hs00183740_m1
SDF-1	chemokine (C-X-C motif) ligand 12	Hs03676656_mH
VEGF	vascular endothelial growth factor A	Hs00900055_m1
IL-8	interleukin 8	Hs00174103_m1
GAPDH	glyceraldehyde-3-phosphate dehydrogenase	Hs02758991_g1

### Western blot

Total protein was isolated from DPC in monolayer cultures after treatment to prepare samples for Western Blot Laemmli Sample Buffer (Bio-Rad Laboratories GmbH, Vienna, Austria) according to the manufacturer’s description and separated on SDS page. After transfer on nitrocellulose membranes, detection was performed using the following primary antibodies (Thermo Fisher Scientific) to detect the target proteins: anti-HIF-1 α antibody (H-206), anti-HIF-2α [EPAS -1 (190b)], and anti-HIF-3α (E-8) (all from Santa Cruz Biotechnology, Santa Cruz, CA, USA). Anti-GAPDH (MA5–15738, Thermo Fischer Scientific) was used to detect the reference protein GAPDH. The primary antibodies were then detected using the appropriate secondary antibody. Then, the chemiluminescence detection was performed with a ChemiDoc MP System (Bio-Rad Laboratories, Inc. CA, USA).

### Enzyme-linked immunosorbent assay

Protein levels of SOST, DKK-1, SDF-1, VEGF and IL-8 were measured by ELISA in collected supernatants from 2D monolayer, 3D spheroid and tooth slice cultures. The procedure was performed according to the manufacturer’s protocols of the respective ELISA kits (SOST, DKK-1: Biomedica Medizinprodukte GmbH & Co KG, Vienna, Vienna, Austria; SDF-1, VEGF, and IL-8: PeproTech Austria, Vienna, Vienna, Austria). Optical density was measured at 450 nm in SOST and DKK-1 ELISA and at 405 nm in SDF-1, VEGF and IL-8 ELISA. Protein concentration was calculated by standard line methods specified by the respective manufacturer.

### Statistics

Statistics were performed with IBM SPSS Statistics Version 23 (IBM Corporation, Armonk, NY, USA), applying the Kruskal-Wallis test and the Mann-Whitney test. The level of significance was set at *p* < 0.05. For monolayer and spheroid cultures N equalled 6 or higher. For tooth slice cultures N equalled 4 if not indicated differently.

## Results

### Cell viability of dental pulp-derived cells in monolayer, spheroid and tooth slice culture after treatment with L-mimosine or hypoxia

**Fig. 1:**
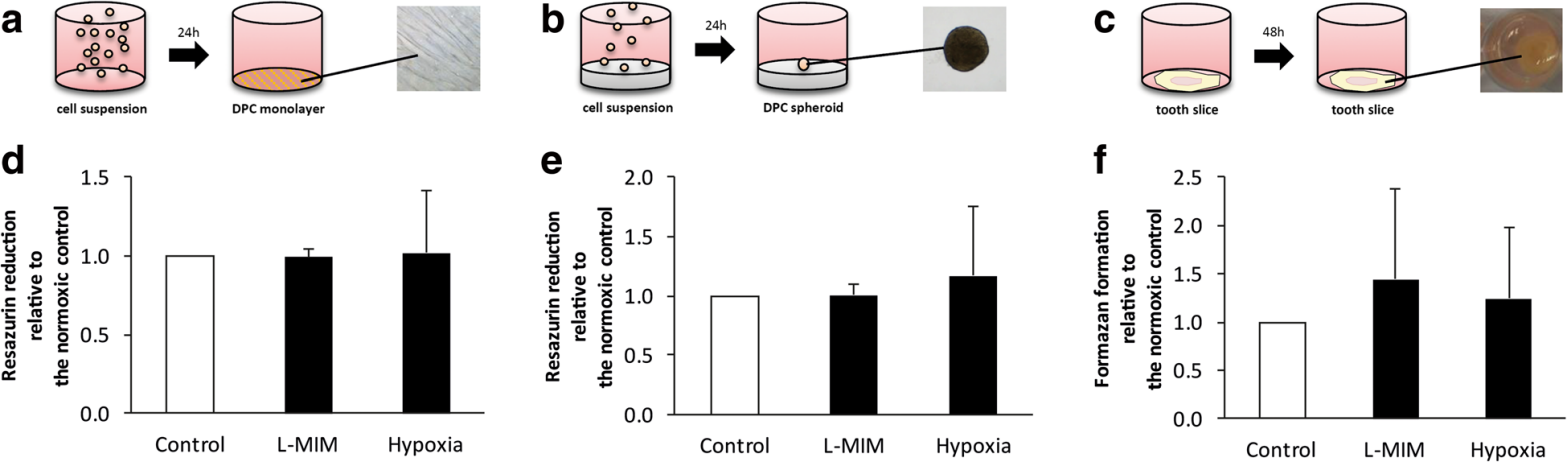
Viability of dental pulp-derived cells in monolayer, spheroid and tooth slice culture upon exposure to L-mimosine (L-MIM) or hypoxia. Dental pulp-derived cells were cultured in 2D monolayer, 3D spheroid and tooth slice cultures to test viability after treatment with 1 mM L-MIM or hypoxia. Cell viability in monolayer (**a**, **d**) and spheroid culture (**b**, **e**) was tested by resazurin-based toxicity assays and pulp viability in tooth slices (**c**, **f**) was tested by MTT assay. Data are displayed as mean ± standard deviation (black bars) and relative to the normoxic control (white bar). The level of significance was set at *p* < 0.05. N equalled 6 or higher

In monolayer cultures (Fig. 1a, d), as well as in spheroid cultures (Fig. 1b, e), DPC appeared to be viable upon treatment with L-MIM or hypoxia in the resazurin-based toxicity assay. There is no significant difference (*p* > 0.05) between metabolic activity of treated cells and the normoxic control. In tooth slice cultures (Fig. 1c, f) reduction of formazan formation in dental pulp tissue treated with L-MIM or hypoxia was not significantly different (*p* > 0.05) from the normoxic control in the MTT assay.

### L-mimosine and hypoxia can stabilize HIF-1α and HIF-2α

**Fig. 2:**
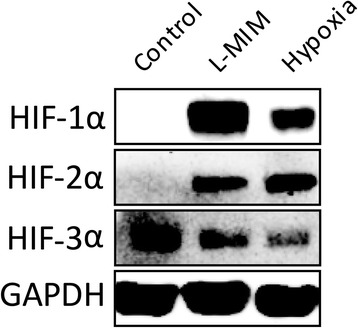
L-mimosine (L-MIM) and hypoxia can stabilize hypoxia-inducible factor (HIF)-1α and HIF-2α. Dental pulp-derived cells were cultured in 2D monolayer and western blots for HIF-1α, HIF-2α and HIF-3α were performed with the appropriate antibodies to validate stabilisation of hypoxia responsive HIFs

Cells were cultured in 2D monolayer and western blots for HIF-1α, HIF-2 α and HIF-3α were performed (Fig. 2). We found bands for HIF-1α, HIF-2α in the L-MIM and hypoxia treated groups and no obvious bands in the untreated control group. For HIF-3α bands were visible in all groups.

### Effects of L-mimosine or hypoxia on the production of sclerostin, dickkopf-1, stromal cell-derived factor-1, vascular endothelial growth factor and interleukin-8 in monolayer cultures of dental pulp-derived cells

The impact of L-MIM and hypoxia on SOST and DKK-1 gene expression and protein production in monolayer cultures of DPC was evaluated in qPCR and ELISA, respectively. To verify the cellular response to hypoxia, the hypoxia-responsive factors SDF-1, VEGF and IL-8 were assessed.

We found SOST (0.00006 ± 0.00010), DKK-1 (0.00453 ± 0.00679), SDF-1 (0.12793 ± 0.29206), VEGF (0.00950 ± 0.02303) and IL-8 (0.00003 ± 0.00004) to be expressed relative to GAPDH in DPC monolayer cultures under normoxic conditions.

**Fig. 3:**
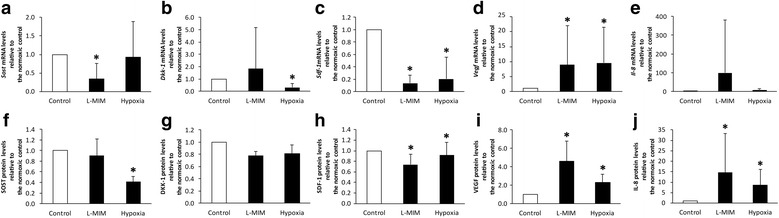
Sclerostin (SOST), dickkopf (DKK)-1, stromal cell-derived factor (SDF)-1, vascular endothelial growth factor (VEGF) and interleukin (IL)-8 upon exposure to L-mimosine (L-MIM) or hypoxia in monolayer cultures. Dental pulp-derived cells were cultured in 2D monolayer culture and treated with 1 mM L-MIM or hypoxia for 24 h. Changes in mRNA levels (*Sost, Dkk-1, Sdf-1, Vegf*, and *Il-8*) were analysed by qPCR (**a**, **b**, **c**, **d**, **e**) and changes in protein levels (SOST, DKK-1, SDF-1, VEGF, and IL-8) were analysed by ELISA (**f**, **g**, **h**, **i**, **j**). Data are displayed as mean ± standard deviation (black bars) and relative to the normoxic control (white bar). The level of significance was set at *p* < 0.05 (*). N equalled 6 or higher

L-MIM significantly downregulated (*p* < 0.05) mRNA expression of SOST (Fig. 3a) and SDF-1 (Fig. 3c), but did not modulate (*p* > 0.05) DKK-1 (Fig. 3b) and IL-8 (Fig. 3e). Hypoxia significantly downregulated (*p* < 0.05) DKK-1 and SDF-1 (Fig. 3c) mRNA expression, while it did not significantly modulate (*p* > 0.05) SOST (Fig. 3a) and IL-8 (Fig. 3e) compared to the normoxic control. VEGF mRNA expression (Fig. 3d) was significantly upregulated (*p* < 0.05) by both, L-MIM and hypoxia treatment relative to the normoxic control.

In supernatants of DPC monolayer cultures, protein production of SOST (18.167 pmol/L ± 7.008), DKK-1 (12.908 pmol/L ± 4.151), SDF-1 (0.741 ng/mL ± 0.555), VEGF (0.502 ng/mL ± 0.361) and IL-8 (0.273 ng/mL ± 0.248) was detectable by ELISA under normoxic conditions.

Treatment with L-MIM significantly downregulated (*p* < 0.05) SDF-1 (Fig. 2h) protein production and upregulated VEGF (Fig. 3i) and IL-8 (Fig. 3j) protein production. Hypoxia significantly downregulated (*p* < 0.05) SOST (Fig. 3f) and SDF-1 (Fig. 3h) protein production and upregulated VEGF (Fig. 3i) and IL-8 (Fig. 3j) significantly (*p* < 0.05). DKK-1 (Fig. 3g) was not significantly modulated (*p* > 0.05) by L-MIM or hypoxia, but showed a slight trend of a decrease upon treatment.

### Effects of L-mimosine or hypoxia on the production of sclerostin, dickkopf-1, stromal cell-derived factor-1, vascular endothelial growth factor and interleukin-8 in spheroid cultures of dental pulp-derived cells

To mimic the 3D situation in the pulp tissue we further evaluated the effect of L-MIM and hypoxia in spheroid cultures of DPC. mRNA expression of SOST (0.00001 ± 0.00001), DKK-1 (0.00029 ± 0.00038), SDF-1 (0.00819 ± 0.00931), VEGF (0.01512 ± 0.00940) and IL-8 (0.00029 ± 0.00049) was detected relative to GAPDH in qPCR in DPC spheroid cultures under normoxic conditions.

**Fig. 4:**
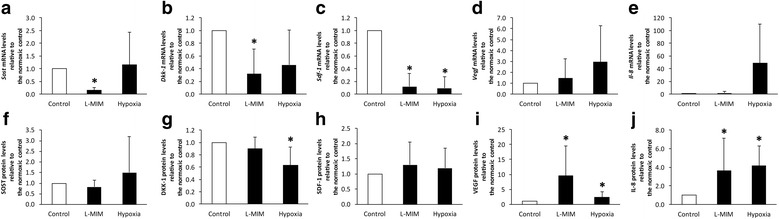
Sclerostin (SOST), dickkopf (DKK)-1, stromal cell-derived factor (SDF)-1, vascular endothelial growth factor (VEGF) and interleukin (IL)-8 upon exposure to L-mimosine (L-MIM) or hypoxia in spheroid cultures. Dental pulp-derived cells were cultured in 3D spheroid culture and treated with 1 mM L-MIM or hypoxia for 24 h. Changes in mRNA levels (*Sost, Dkk-1, Sdf-1, Vegf,* and *Il-8*) were analysed by qPCR (**a**, **b**, **c**, **d**, **e**) and changes in protein levels (SOST, DKK-1, SDF-1, VEGF, and IL-8) were analysed by ELISA (**f**, **g**, **h**, **i**, **j**). Data are displayed as mean ± standard deviation (black bars) and relative to the normoxic control (white bar). The level of significance was set at *p* < 0.05 (*). N equalled 6 or higher

In DPC spheroid cultures, L-MIM significantly downregulated (*p* < 0.05) SOST (Fig. 4a), DKK-1 (Fig. 4b) and SDF-1 (Fig. 4c) at mRNA levels, while hypoxia only reduced SDF-1 (Fig. 4c) (*p* < 0.05). VEGF (Fig. 4d) and IL-8 (Fig. 4e) mRNA expression is not significantly modulated (*p* > 0.05) by L-MIM or hypoxia in DPC spheroids, but shows a trend to increase upon treatment with hypoxia.

In DPC spheroid culture supernatants SOST (1.530 pmol/L ± 0.391), DKK-1 (1.568 pmol/L ± 0.562), SDF-1 (0.165 ng/mL ± 0.082), VEGF (1.349 ng/mL ± 1.074) and IL-8 (0.006 ng/ mL ± 0.003) protein levels were measured by ELISA under normoxic conditions.

In DPC spheroid cultures, VEGF (Fig. 4i) and IL-8 (Fig. 4j) production was significantly upregulated (*p* < 0.05) by L-MIM and hypoxia. Hypoxia also significantly downregulated (*p* < 0.05) DKK-1 (Fig. 4g) protein production in the 3D DPC model. SOST and SDF-1 were not modulated significantly.

### Effects of L-mimosine and hypoxia on the production of sclerostin, dickkopf-1, stromal cell-derived factor-1, vascular endothelial growth factor, and interleukin-8 in tooth slice cultures

To mimic the in vivo situation in the dentine-pulp complex we evaluated the impact of L-MIM and hypoxia in tooth slice cultures. In dental pulp tissue of tooth slices, SOST (0.00118), DKK-1 (0.00030 ± 0.00039), SDF-1 (0.03475 ± 0.03710) and IL-8 (0.02612 ± 0.00930) were expressed relatively to the GAPDH under normoxic conditions. SOST mRNA levels of all treatment groups were only detected in samples of one donor tooth and DKK-1 mRNA was only found in normoxic controls. Tooth slices treated with L-MIM or hypoxia showed a tendency for a decrease in SOST which reached the level of 0.102-fold and 0.244-fold, respectively. No pronounced impact of L-MIM and hypoxia on VEGF was found at mRNA levels which were 0.930-fold and 0.773-fold of the normoxic control, respectively. SDF-1 showed a tendency to decrease and reached 0.323-fold and 0.162-fold of the normoxic control. IL-8 mRNA levels tend to be decreased by treatment with L-MIM were they reached 0.445-fold of the normoxic control while treatment with hypoxia showed a trend for an increase with 1.774-fold of the normoxic control. Due to low donor tooth availability, no statistical evaluation was performed for these results.

In supernatants of tooth slice cultures with dental pulp tissue under normoxic conditions, SOST (2.359 pmol/L ± 1.035), DKK-1 (2.332 pmol/L ± 0.406), VEGF (1.046 ng/mL ± 0.626) and IL-8 (2.043 ng/mL ± 1.396) protein levels were measureable by ELISA. SDF-1 could not be measured in any of the tooth slice supernatants as the protein levels were below the detection limit.

The absolut concentration in the normoxic control group and the treatment groups was normalised to the respective MTT results to correct for the volume of metabolic active tissue and is presented relative to the corresponding normoxic control. After treatment with L-MIM or hypoxia SOST protein levels were 1.433-fold and 1.236-fold of the normoxic control, respectively. DKK-1 protein levels were 1.035-fold and 1.101-fold of the normoxic control for L-MIM and hypoxia, respectively. VEGF protein levels were 1.585-fold and 1.513-fold of the normoxic control for L-MIM and hypoxia, respectively. IL-8 protein levels were 0.888-fold and 1.447-fold of the normoxic control for L-MIM and hypoxia, respectively.

## Discussion

In this work the effects of L-MIM and hypoxia on the production of the WNT signalling inhibitors SOST and DKK-1 were analysed in human DPC. The cells stayed vital and elevated levels of HIF-1α and HIF-2α were observed upon incubation with L-MIM and hypoxia. Overall, the levels of SOST and DKK-1 were low in all used models: the monolayer cultures, spheroid cultures and tooth slice cultures. In monolayer cultures, the levels of SOST and DKK-1 mRNA were downregulated by L-MIM and hypoxia, respectively. Hypoxia significantly downregulated SOST at the protein level compared to untreated cells while the effect of hypoxia on DKK-1 and the impact of L-MIM on SOST and DKK-1 did not reach the level of significance. In spheroid cultures, mRNA levels of SOST and DKK-1 were downregulated by L-MIM while a significant downregulation of DKK-1 protein upon hypoxia treatment was found. The impact of hypoxia on SOST protein levels and the effect of L-MIM on SOST and DKK-1 protein levels did not reach the level of significance. Our results show that L-MIM and hypoxia treatments have different effects at mRNA and protein levels within each model. The induction of a hypoxia-like response was validated by increased VEGF and IL-8 production and elevated levels of HIF-1α and HIF-2α in DPC upon treatment with hypoxia or L-MIM. This is in line with our previous findings in DPC monolayer cultures and DPC during spheroid formation [6, 7, 8, 24, 25]. Based on the current literature it can be suggested that this increase in pro-angiogenic factors is based on the stabilisation of HIF isoforms. Also SDF-1 has been reported to be produced in dental pulp cells [30] and to be involved in chemotaxis [31], inflammation [32] and odontoblast differentiation [33]. SDF-1 is downregulated by L-MIM and hypoxia at mRNA levels in 2D monolayer cultures and 3D spheroid cultures of DPC, while the protein levels only marginally decrease in monolayers of DPC. These results suggest no strong impact of hypoxia and L-MIM on SDF-1 production in DPC. Interestingly, in fibroblasts of other tissues hypoxia stimulates SDF-1 production [34].

Highly increased levels of SOST and DKK-1 are associated with compromised hard tissue formation [35, 36], while strong reduction of SOST and DKK-1 is associated with a strong induction of hard tissue formation [37–39]. In case of pulp healing it may not be beneficial to induce either of them as it leads to pulp calcification. It is unclear if the low SOST and DKK-1 in particular upon L-MIM or hypoxia treatment are sufficient to inhibit WNT signalling in dental pulp cells. Our data suggest that hypoxia and hypoxia mimetic agents do not induce a strong anti-WNT signalling response in the dental pulp cells under basal conditions. Thus, hypoxia and hypoxia mimetic agents are not expected to compromise the cellular response with regards to the tightly controlled production of WNT inhibitors SOST and DKK-1.

It is interesting that the mRNA and protein levels in particular for SOST and DKK-1 do not directly parallel each other. This difference in mRNA and protein levels might be due to transcriptional and/or translational regulation of SOST and DKK-1. Also the fact that mRNA of SOST and DKK-1 of lysed cells were assessed and SOST and DKK-1 protein levels were evaluated in the released supernatant of the cells by ELISA and not intracellular protein by western blotting might contribute to the differences. Future studies are required to shed light on this issue.

In our study we evaluated the impact of L-MIM and hypoxia on SOST and DKK-1 under basal conditions. For hypoxia incubation we used a pouch system which mimics severe hypoxia < 1% O_2_. These levels were also suggested by Agata et al. [26] as biologically relevant [1, 2]. However, in previous studies a dominant increase of SOST and DKK-1 production was observed upon treatment with transforming growth factor (TGF)-β [40] and interleukin (IL)-1 [41], respectively. It is possible that the trend of a reduced SOST and DKK-1 production upon treatment with L-MIM observed in basal conditions translates into an inhibition of the induction of SOST and DKK-1 by TGF-β and IL-1, respectively.

In this work we used three different culture models of which each has its advantages and disadvantages. The traditional monolayer culture is a suitable model to analyse if cells respond to certain treatments or conditions and also numerous assays and methods allow to analyse them in detail. Nevertheless, monolayers of cells on plastic surfaces do not reflect the in vivo situation by far as they also manipulate cell morphology, differentiation or gene expression [42]. Spheroid cultures in contrast provide a situation which overcomes these aspects more likely. Our results reflect that there is a difference in mRNA and protein production of the same genes and proteins, depending on the model. The data show that L-MIM and hypoxia differently affect DPC in 2D and 3D cultures and even if the same effects or trends can be seen in both the models, intensities vary. We used a heterogeneous cell population, since cells were not characterised after isolation from the pulpal tissue. However, it can be assumed that both culture systems are based on predominantly fibroblasts due to the higher proliferation rate in the expansion process. The tooth slice culture represents the dentine-pulp complex of a vital tooth more closely and is thus a more complex setting due to the complex tissue structure. Results from tooth slice cultures demonstrate that in this model, where the in vivo environment is mimicked even more closely than in the 3D culture, no modulations of SOST and DKK-1 can be detected after treatment with L-MIM or hypoxia. It is possible that extraction and the cutting process can have an impact on the production of SOST and DKK-1 and thus interfere in this system. Also hypoxic zones might develop in the tooth slices decreasing the responsiveness of the tissue to additional treatment with hypoxia or hypoxia mimetic agents. The fact that the pulp tissue is responsive to treatment with hypoxia mimetic agents however does not support this assumption [7, 8, 25, 29]. This shows the importance to develop more in vivo-like in vitro models to avoid unnecessary pre-clinical studies in the course of therapy development.

## Conclusions

In conclusion it can be stated that there is no pronounced influence of hypoxia and L-mimosine on DPC viability, SOST and DKK-1 protein production. Thereby, this study deepens our understanding on potential impacts of hypoxia on dental pulp.

## Abbreviations

αMEM: Alpha minimum essential media
DKK-1: Dickkopf-1
DNA: Deoxyribonucleic acid
DPC: Dental pulp-derived cells
ELISA: Enzyme-linked immunosorbent assays
FBS: Foetal bovine serum
GAPDH: Glycerinaldehyde-3-phosphate dehydrogenase
HIF: Hypoxia-inducible factor
IL-8: Interleukin-8
L-MIM: L-mimosine
mRNA: Messenger ribonucleic acid
PCR: Polymerase chain reaction
qPCR: Quantitative polymerase chain reaction
RNA: Ribonucleic acid
SDF-1: Stromal cell-derived factor-1
SOST: Sclerostin
TGF-β: Transforming growth factor-beta
VEGF: Vascular endothelial growth factor
